# Microbial DNA analysis of paired blood-bronchoalveolar lavage fluid in post-HSCT patients with pneumonia implying application conditions of blood as a surrogate in pathogen detection

**DOI:** 10.1186/s12931-026-03779-z

**Published:** 2026-06-18

**Authors:** Ruiqi Li, Xiangyan He, Zhonglin Chen, Jie Shao, Jiangzhou Feng, Liping Wan, Mengyuan Zhang, Jun Yang, Yin Tong, Baoxia Dong, Chongmei Huang, Huiying Qiu, Yu Cai, Jiahua Niu, Xiaowei Xu, Xianming Song, Jinmin Ma, Hongfeng Ge, Kun Zhou

**Affiliations:** 1 Department of Hematology, Shanghai general hospital, Shanghai Jiao Tong University School of Medicine, Shanghai, 200080 China; 2 BGI Huo-Yan Engineering Technology, Shenzhen, 518083 China; 3 Department of Hematology, Bozhou Hospital Affiliated to Anhui Medical University, Anhui, 236800 China

**Keywords:** Paired blood-bronchoalveolar lavage fluid, Immunocompromised pneumonia patients, Metagenomic, Transcriptomic, Pathogen detection

## Abstract

**Background:**

Blood testing aids pneumonia diagnosis, but its effectiveness varies. Given the invasiveness of bronchoalveolar lavage fluid (BALF) sampling versus blood testing’s simplicity, this study investigates when blood can reliably substitute for BALF in detecting microbial presence, especially for pathogens.

**Results:**

Metagenomic sequencing was performed on paired BALF-blood samples from 21 post-HSCT immunocompromised (ICP) and 21 immunocompetent (ICT) patients. The ICP cohort was expanded to 62 for biomarker validation. Host responses were profiled via metatranscriptomics (30 BALF samples). Microbial alpha and beta diversity differed significantly between blood and BALF in ICP, but not ICT, patients. ICP patients’ BALF contained a greater diversity and abundance of microbes. A higher proportion of microbial DNA sequences in ICP patients’ blood was also present in their BALF, suggesting a potentially more permeable alveolar-capillary barrier. Related genes (e.g., NABA CORE MATRISOME, extracellular matrix organization, cell-cell adhesion) were downregulated. Upregulated pathways like VEGFA-VEGFR2 signaling and Rho GTPases suggested increased vascular permeability. In ICP patients, 419 microbial sequences in blood indicated their presence in the lower respiratory tract with > 70% certainty.

**Conclusion:**

Host immune status significantly influences blood-BALF microbial diversity differences. Shared blood-BALF microbial DNA sequences show potential for aiding pneumonia pathogen diagnosis, offering a novel biomarker identification approach.

**Supplementary Information:**

The online version contains supplementary material available at https://doi.org/10.1186/s12931-026-03779-z.

## Background

Studies involving large cohorts suggested that healthy individuals do not have a consistent blood microbiome, with detected microbial species likely originating from transient translocation from the gut, oral cavity, skin, or respiratory tract [1]. However, in diseased states, is there any increased microbial translocation into the blood? Numerous studies have demonstrated that blood or plasma metagenomic next-generation sequencing (mNGS) can effectively detect pneumonia pathogens [2–4], possibly due to either pathogen migration into the bloodstream or circulating microbial DNA.

Compared to bronchoalveolar lavage fluid (BALF) sampling, blood collection is far less invasive, particularly for immunocompromised (ICP) or critically ill pneumonia patients. However, current studies [3, 5–7] indicate that the sensitivity of blood/plasma mNGS in diagnosing pulmonary infections is only 25–50%, whereas BALF mNGS sensitivity ranges from 81.3% to 100%, highlighting a low agreement between blood and BALF testing. Some studies [4, 8] report improved blood mNGS sensitivity (approximately 70%) when pneumonia progresses to sepsis, suggesting its potential utility in such cases. Additionally, plasma mNGS may be particularly effective for detecting viral pneumonia pathogens [3, 5]. To capitalize on the convenience of blood testing while maximizing its diagnostic value, it is crucial to identify specific clinical scenarios where blood testing could substitute BALF testing.

Understanding how microorganisms cross from the lower respiratory tract into the bloodstream—whether in terms of microbial types or concentration—is crucial for determining the feasibility and conditions of using blood tests as a surrogate to diagnose pulmonary infections. However, there is currently no literature addressing this specific area of research.

This study aims to elucidate the conditions under which blood test results can reliably diagnose pulmonary infections by comparing microbial sequences, community composition, and abundance between BALF and blood samples from ICP pneumonia patients. We will specifically identify which blood-detected microorganisms reliably indicate lung infection. Furthermore, we will analyse paired BALF and blood samples from immunocompetent (ICT) patients to investigate how immune statuses affect these findings. To our knowledge, this represents the first comprehensive comparison of microbial profiles between paired blood and BALF samples from both ICP and ICT patients.

## Method

### Study design

This study included two cohorts: a discovery cohort and a validation cohort. For the discovery cohort, 21 patients with a history of allogeneic hematopoietic stem cell transplantation (HSCT) and pneumonia were prospectively enrolled at Shanghai First People’s Hospital between 2021 and 2023. ICP status was defined according to the 2013 and 2025 Infectious Diseases Society of America (IDSA) guidelines as meeting any of the following: (i) CD4 + T-cell count < 200/µL or CD4% <15%; (ii) absolute neutrophil count < 500/µL; (iii) serum IgG < 400 mg/dL; (iv) continuous high-dose glucocorticoids (≥ 20 mg/day prednisone equivalent) or other immunosuppressants for ≥ 14 days within the past 3 months. All enrolled HSCT patients met at least one of these criteria and were designated as the ICP group. For comparison, 21 immunocompetent ICT patients with pneumonia were also included from a previous study [9]. These ICT patients had no history of allogeneic HSCT, no other immunocompromising conditions (e.g., solid organ transplantation, long‑term corticosteroid or biologic use, primary immunodeficiency), and no significant comorbidities such as diabetes, hypertension, chronic liver or kidney disease, autoimmune disorders, or other malignancies. They were otherwise previously healthy individuals. Paired blood and BALF samples were collected from each participant within 24 h. For the validation cohort, an additional 41 HSCT patients with pneumonia meeting the same ICP criteria were enrolled, totaling 62 ICP patients. Validation samples underwent deeper sequencing with approximately 10-fold increased depth.

Inclusion criteria for pneumonia were: (1) fulfillment of the above ICP criteria (or no immune defects for ICT); (2) abnormal chest X‑ray defined as new infiltrates, consolidation, ground‑glass opacities, or nodules with a halo sign (excluding isolated pleural thickening, old fibrotic lesions, atelectasis, or effusion without parenchymal involvement); (3) at least two respiratory symptoms (e.g., fever, cough, dyspnea) and elevated peripheral blood inflammatory markers (C‑reactive protein, procalcitonin) above the upper limit of normal; (4) final clinical diagnosis of infectious pneumonia confirmed by comprehensive evaluation, including microbiological tests (blood cultures, fungal G/GM tests, respiratory virus PCR, BALF bacterial/fungal cultures, *Pneumocystis jirovecii* nucleic acid testing) and response to anti‑infective therapy; (5) successful collection of blood and BALF samples within 24 h, with BALF obtained before initiation of anti‑infective therapy. Exclusion criteria were: (1) failure to obtain paired samples within 24 h; (2) incomplete clinical data; (3) confirmed non‑infectious pneumonia.

All patients provided written informed consent. The study was approved by the Institutional Review Board of Shanghai First People’s Hospital (No. 2018KY270).

**Fig. 1:**
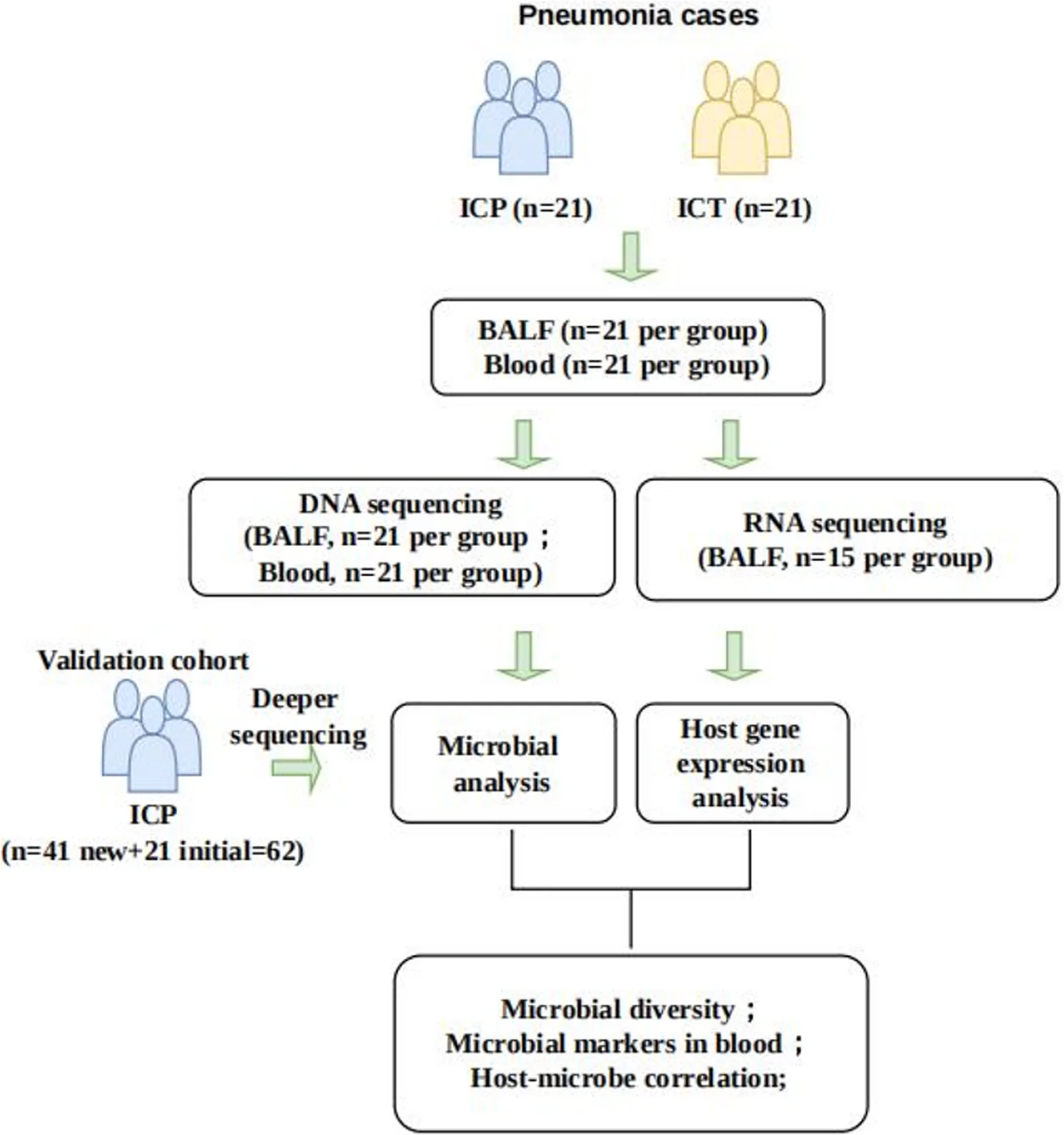
Study design. ICP: immunocompromised patients; ICT: immunocompetent patients; BALF: bronchoalveolar lavage fluid. The initial cohort comprised 21 paired ICP and ICT subjects. To enhance statistical power, an additional 41 ICP patients were enrolled for validation. *The 21 initial ICP patients were from the discovery cohort

Blood and BALF samples were collected from each patient and were subjected to mNGS to investigate the differences in microbial sequences between the two sample types and the two patient groups (Fig. 1). Besides, to explore the potential host mechanism in microbial variation, fifteen BALF samples from each group were applied for RNA sequencing.

### Clinical comprehensive diagnosis

The definitive pathogen identification was established through a comprehensive diagnostic approach integrating: (1) microbiological confirmation via culture or nucleic acid amplification techniques (including PCR and mNGS) of paired blood and BALF samples, with quantitative real‑time PCR for cytomegalovirus (CMV)‑DNA and Epstein-Barr virus (EBV)‑DNA as part of viral nucleic acid testing; (2) serological profiling, including the G test (1,3‑β‑D‑glucan), GM test (galactomannan), and detection of IgM antibodies against common respiratory pathogens such as Mycoplasma pneumoniae, Chlamydia pneumoniae, Legionella pneumophila, and respiratory viruses (e.g., influenza, adenovirus); (3) assessment of host inflammatory biomarkers, specifically C‑reactive protein (CRP), procalcitonin (PCT), endotoxin, and erythrocyte sedimentation rate (ESR); (4) evaluation of clinical manifestations and therapeutic response patterns, integrating symptoms, imaging findings, and response to empirical anti‑infective therapy (e.g., improvement in fever, respiratory symptoms, or radiographic abnormalities after targeted or broad‑spectrum antibiotic/antifungal/antiviral treatment). All diagnostic conclusions underwent rigorous validation by two independent senior clinicians to ensure diagnostic accuracy.

### Sample collection and nucleic extraction

BALF (5 mL) and blood (3 mL) samples were collected from ICP patients according to standard procedures and pretreated before further nucleic acid extraction [9, 10]. The sample treatment and nucleic extraction procedures followed the protocol used in a previous study 9. The extracted DNA and RNA were quantified by Qubit and subsequently employed for DNA library construction. For RNA samples, the complementary DNA (cDNA) was transcribed and then the secondary DNA strand was synthesised prior to the generation of the DNA library.

### Library construction and sequencing

The DNA library was constructed as described in the previous study [9] and involved four main steps: DNA fragmentation, end-repair, adapter-ligation and PCR amplification. The constructed double-strand DNA library was assessed by Agilent 2100 (Agilent Technologies, Santa Clara, CA) and Qubit 2.0 (Invitrogen, USA). Then, a single-stranded circular DNA library was generated through DNA denaturation and circularization of the qualified double-strand DNA library. Subsequently, through rolling circle amplification (RCA), DNA nanoballs (DNBs) were formed. Finally, the qualified DNBs were sequenced on the MGISEQ-2000 platform (MGI, China).

### Quality control on DNA sequencing data and microbial sequence analysis

The produced raw sequencing data were initially filtered with fastp to eliminate low-quality reads and adapters before undergoing further assessment with FastQC (Version 0.11.9) [11, 12]. Next, to remove human sequences, the filtered and qualified reads were mapped to the human genome (GRCh38) using HISAT2 (2.2.1 release) [13]. The remaining sequences were identified by Kraken2 [14].

To mitigate potential contaminations, the negative control and samples were processed with R package decontam [15]. Furthermore, to specifically address possible gut-derived microbial DNA contamination in blood samples, microbial DNA reads were first identified using MetaPhlAn 3 (v3.0) within the bioBakery 3 platform’s standardized analytical pipeline [16], followed by their systematic removal from the blood sample datasets. This rigorous filtering process ensured that the remaining microbial DNA reads in blood samples predominantly originated from BALF rather than intestinal sources. Consequently, this approach significantly enhanced the accuracy of the identified microbial markers in blood, thereby providing a more reliable representation of their presence in BALF.

Principal component analysis (PCA) was conducted with ade4 R package [17]. Microbial diversity including α-diversity and β-diversity was analysed by R package Fossil [18] and VEGAN [19] respectively. To determine the differently abundant microbes between two groups, the foldchange of abundance and statistics were analysed by DEseq2 package [20]. The criteria for significant difference were log2|FoldChange| >= 1 & padj < 0.05. The figures including PCA plots, box plots and heatmap were produced using the ggplot2 package in R v4.0.3 [21]. The Veen plots were generated by online program EVenn [22].

### Quality control on RNA sequencing data and differential gene expression analysis

The RNA-seq raw data were filtered and quality controlled as the DNA-seq data. Then the filtered reads were mapped to the human genome (GRCh38) using hisat2 (version 2.2.1), and reads per kilobase per million mapped reads (RPKM) values for host gene expression were calculated with the “edgeR” R package. The genes detected in less than eight samples in ICP or ICT were removed. Differentially expressed genes (DEGs) between two groups were analyzed using RStudio (version 1.3.959) with the DESeq2 package. DEGs were defined as those with an absolute fold change greater than 2 and an adjusted p-value < 0.01. DEGs were displayed in a volcano plot and a heatmap, and were also subjected to functional enrichment analysis conducted on the Metascape website [23].

### The development of a classifier

We aimed to identify microbial markers that could effectively distinguish between ICT and ICP. The identified microbes, represented by their relative abundance (Reads Per Million), were initially considered as potential markers. To assess the significance of each microbe, we employed the Random Forest Regressor in Python (version 3.8.8). Using the top 20 microbes, we experimented with ten different machine-learning methods to construct a classifier. The performance of each model was evaluated using ROC curves, and the model demonstrating the best performance was selected as the final classifier.

### Statistical analysis

The statistical significance of differences between groups in PCA plot was tested by pairwise permutational multivariate analysis of variation (PERMANOVA). The comparisons of microbial diversity between groups were examined by Wilcoxon rank sum test. The significance of differences in clinical characteristics between the two groups was obtained by wilcox.test() in Rstudio (R version 4.4.1). Spearman correlation analysis was employed to assess associations between microbial features, host gene expression, and clinical parameters. Correlation strengths were interpreted as follows: |ρ| ≥ 0.6 indicated strong associations, while |ρ| ≥ 0.8 denoted very strong associations [24, 25].

## Results

### Patient characteristics, clinical diagnostic results, and sequencing data

The initial study cohort comprised 21 ICP patients, (14 male,7 female; mean age of 52.19 ± 12.42 years) and 21 ICT controls (15 male, 6 female; mean age of 53.52 ± 13.84 years), with no significant age difference (*p* = 0.748, Student’s t-test). As detailed in Supplementary Table 1, ICP patients exhibited significantly altered clinical parameters including decreased red/white blood cell counts, liver enzymes (ALT, alanine aminotransferase; AST, aspartate aminotransferase; LDH, lactate dehydrogenase), renal marker (BUN, blood urea nitrogen), and inflammatory indicators (CRP, C-reactive protein; PCT, procalcitonin) compared to ICT controls. Apart from the underlying hematologic disorders that necessitated HSCT in the ICP group, no patient in either group had other significant comorbidities, except for one ICP patient who had well‑controlled hypertension. All ICT patients had no such comorbidities. Notably, both white blood cell counts and CD4 T cell percentages were significantly lower in ICP patients.

Based on comprehensive clinical diagnosis integrating conventional microbiological tests, mNGS results from both blood and BALF, and clinical response to therapy, 14 pathogen species were identified in the ICP group,, including 5 viral, 5 fungal and 4 bacterial species. In contrast, in the ICT group, 11 species were identified, only consisting of 8 bacterial and 3 viral species, with no fungal pathogen found. Detailed information on the pathogens is presented in Supplementary Table 2. Notably, no consistent pathogens were found in the two groups indicating an obvious difference in pathogen profile between them.

For microbiome comparison, paired blood-BALF samples were collected from all patients within 24 h and subjected to DNA sequencing. The sequencing depths were consistent between groups for each sample type. The mean clean sequencing data for blood samples was 39.05 ± 2.28 M reads for the ICP group and 38.40 ± 1.95 M reads for the ICT group (Supplementary Table 3). For BALF samples, the ICP group had 23.97 ± 6.14 million reads, while the ICT group had 23.23 ± 1.43 million reads. Interestingly, the ratios of microbial reads to total clean reads were 0.092% in blood and 1.578% in BALF for ICP patients, compared to 0.140% in blood and 0.142% in BALF for ICT patients. The percentage of microbial reads was significantly higher in the BALF compared to blood in ICP patients, while it remained similar in ICT patients. Notably, the microbial reads in the BALF of ICP patients were 12 times higher than those in ICT patients, suggesting a greater microbial abundance in the BALF of ICP patients.

### Analysis and comparison of the overall microbial composition and diversity of blood and BALF

**Fig. 2:**
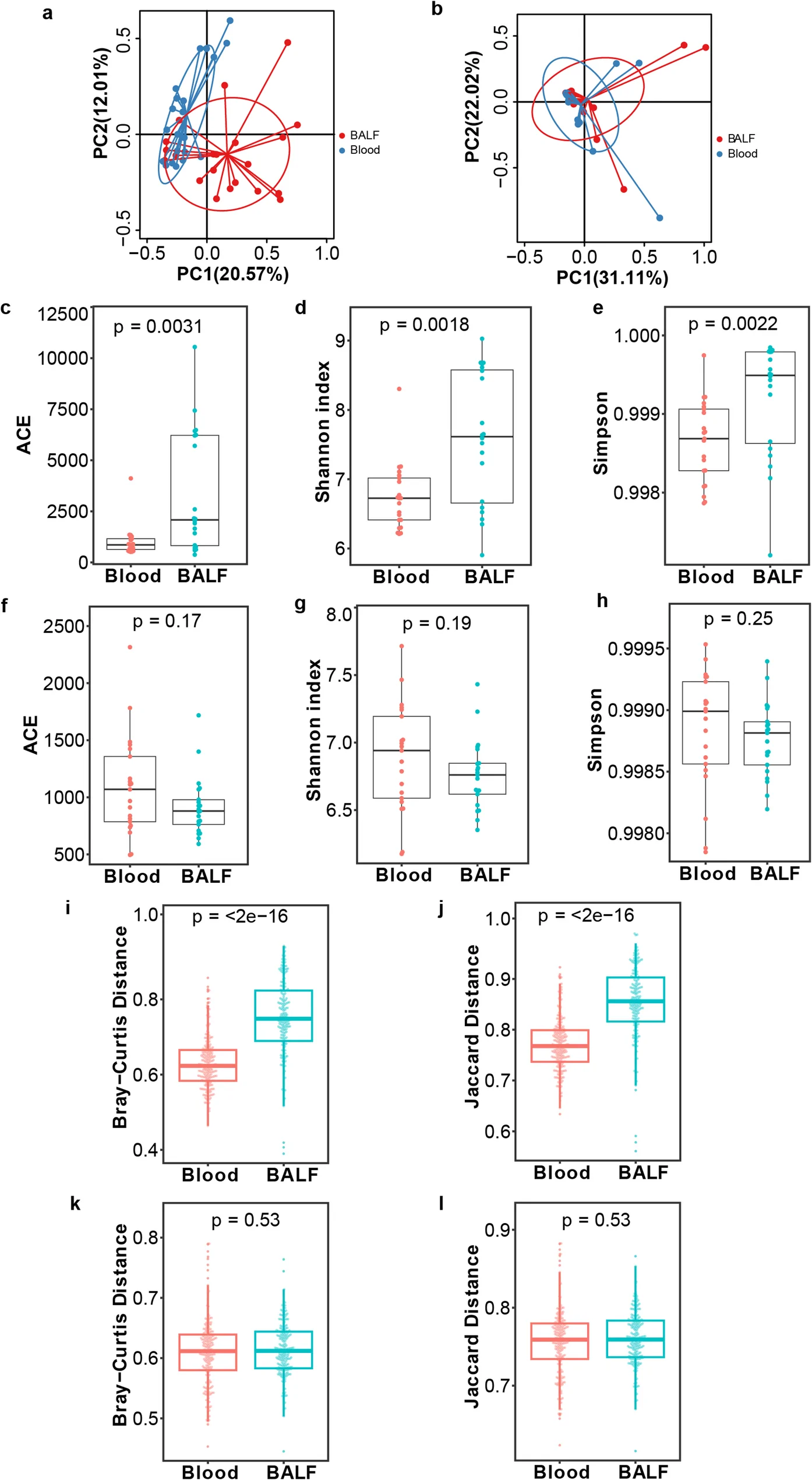
Comparative analysis of microbial DNA composition and diversity in blood versus BALF. **a**-**b** Principal component analysis (PCA) of microbial communities based on Bray-Curtis distances showing significant separation between blood (blue dot) and BALF (red dot) samples from (**a**) 21 ICP and (**b**) 21 ICT patients (PERMANOVA). **c**-**h** Box plots comparing alpha diversity between matched blood-BALF pairs: **c**-**e** ACE, Shannon, and Simpson indices in ICP cohort; **f**-**h** Corresponding indices in ICT cohort. Significance determined by paired Wilcoxon signed-rank test. **i**-**l** Beta diversity dissimilarity metrics: **i**-**j** Bray-Curtis and Jaccard distances for ICP group; **k**-**l** Corresponding distances for ICT group. Center lines represent medians, boxes show IQR (paired Wilcoxon test)

To visualize differences in overall microbial composition, we performed principal component analysis (PCA). The results revealed significant differences in microbial composition between blood and BALF samples in ICP group (*P* = 0.001, PERMANOVA) and ICT group (*P* = 0.04, PERMANOVA) (Fig. 2a and b). Furthermore, distinct microbial diversity patterns were observed between ICP and ICT patients for both blood (*P* = 0.001, PERMANOVA) and BALF samples (*P* = 0.001, PERMANOVA) (Supplementary Fig. 1a, 1b).

Further analysis of microbial alpha-diversity showed significantly higher diversity in BALF compared to blood in ICP patients (Fig. 2c-e), including ACE index (*P* = 0.0031, Wilcoxon signed-rank test), Shannon index (*P* = 0.0018, Wilcoxon signed-rank test) and Simpson index (*P* = 0.0022, Wilcoxon signed-rank test). In contrast, the diversity in ICT patients was similar between blood and BALF, with no significant difference (Fig. 2f-h). The alpha-diversity for BALF was significantly higher in ICP than ICT patients, whereas those for blood were comparable between them (Supplementary Fig. 1c-h). The analysis of microbial beta-diversity between BALF and blood in ICP and ICT patients displayed the same trends as their alpha-diversity (Fig. 2i-l). The microbial beta-diversity in BALF and blood was distinctly higher in ICP patients compared to ICT patients (Supplementary Fig. 1i-l).

### Analysis of differential microbial profiles in blood and BALF and their correlation with disease

**Fig. 3:**
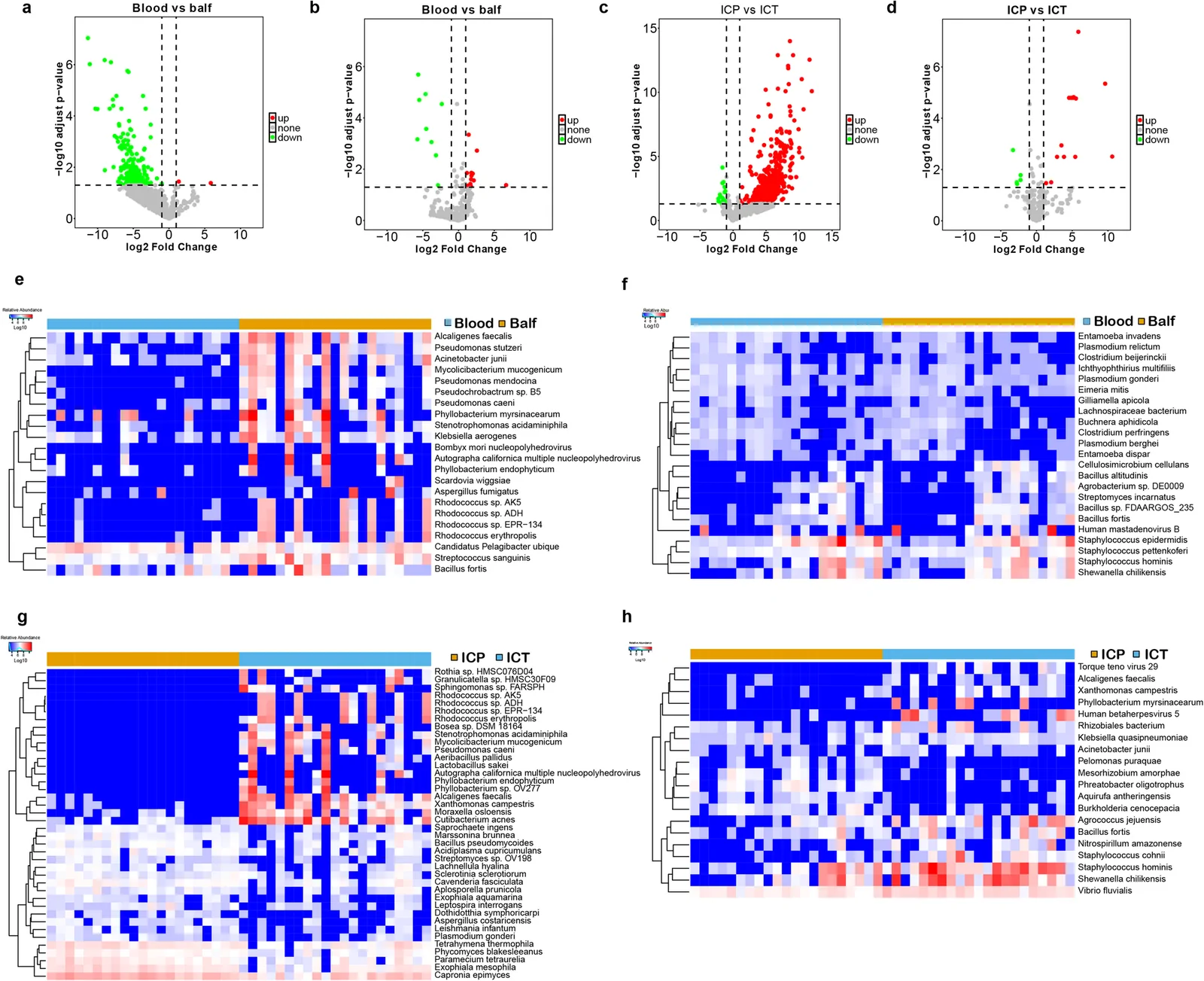
Differential microbial DNA profiles between blood and BALF compartments. **a**-**b** Volcano plots showing significantly enriched (red; log2FC >1, FDR <0.05) and depleted (green; log2FC <-1, FDR <0.05) microbial species in: **a** blood vs. BALF from ICP patients; **b** blood vs. BALF from ICT patients. **c**-**d** Species-level differences between patient groups: **c** BALF of ICP vs. ICT patients; **d** blood of ICP vs. ICT patients. **e**-**f** Heatmaps of the 15 most enriched and depleted microbial species in blood relative to matched BALF from: **e** ICP cohort (n=21); **f** ICT cohort (*n*=21). **g**-**h** Heatmaps displaying top 15 upregulated and downregulated species in: **g** ICP vs. ICT BALF samples; **h** ICP vs. ICT blood samples

Further analysis of differential microbial abundance between blood and BALF samples from ICP patients and ICT patients revealed distinct patterns. In ICP patients, two microbial species showed significantly higher abundance in blood compared to BALF while 249 species were more abundant in BALF. The ICT group demonstrated a different profile, with 13 species more abundant in blood and 10 in BALF (Fig. 3a and b, Supplementary Tables 4,5). Notably, the blood-enriched microbial species were entirely distinct between ICP and ICT groups (Fig. 3c and d, Supplementary Table 6). Only four bacterial species including *Bacillus fortis*,* Staphylococcus pettenkoferi*,* Bacillus altitudinis*, and *Agrobacterium* sp. DE0009 consistently exhibited higher abundances in BALF across both groups. Comparative analysis between ICP and ICT patients identified 859 upregulated and 20 downregulated microbial species in BALF (Supplementary Table 7), in contrast to only 15 upregulated and 5 downregulated species in blood. These findings indicate the profound influence of immune status on the lower respiratory tract microbiome. Conversely, these microbial signatures may serve as valuable discriminative markers of immune status. We assessed the performance of these microbial markers to distinguish ICT from ICP patients using a Random Forest Regressor model. The resulting classifier, which incorporated 20 microbes—including *Xanthomonas campestris*,* Cutibacterium acnes*, and *Pseudomonas putida*—demonstrated impressive performance, achieving an area under the curve (AUC) of 0.91 (Supplementary Fig. 2, Supplementary Table 8). Of these, 14 microbes were significantly higher in abundance in ICP patients. Such markers may provide crucial insights for risk stratification and clinical management in ICP populations, potentially guiding enhanced supportive care and closer monitoring to ultimately improve patient outcomes. Furthermore, the upregulated microbes in BALF from ICP patients compared to the ICT patients were significantly associated with several diseases such as rheumatoid arthritis (*p* = 2.12E-7), cirrhosis (*p* = 0.004), colorectal cancer (*p* = 0.013), enterocolitis (*p* = 0.033) according to MicrobiomeAnalyst online platform [26] (Supplementary Fig. 3).

Heatmaps were generated to visualize microbial abundance across samples, displaying the top 15 abundant microbes (Fig. 3e-h). Consistent with the volcano plot results, BALF samples from ICP patients showed higher microbial abundance compared to blood samples, with these microbes being particularly enriched in most samples. This pattern indicates extensive microbial colonization in the lower respiratory tract of ICP patients, a phenomenon not observed in ICT patients. The top 15 upregulated microbes in BALF from the ICP group showed remarkably different abundances compared to the ICT group, highlighting their potential as biomarkers for immune status differentiation.

### Comparative analysis of shared and specific microbial composition in blood and BALF

**Fig. 4:**
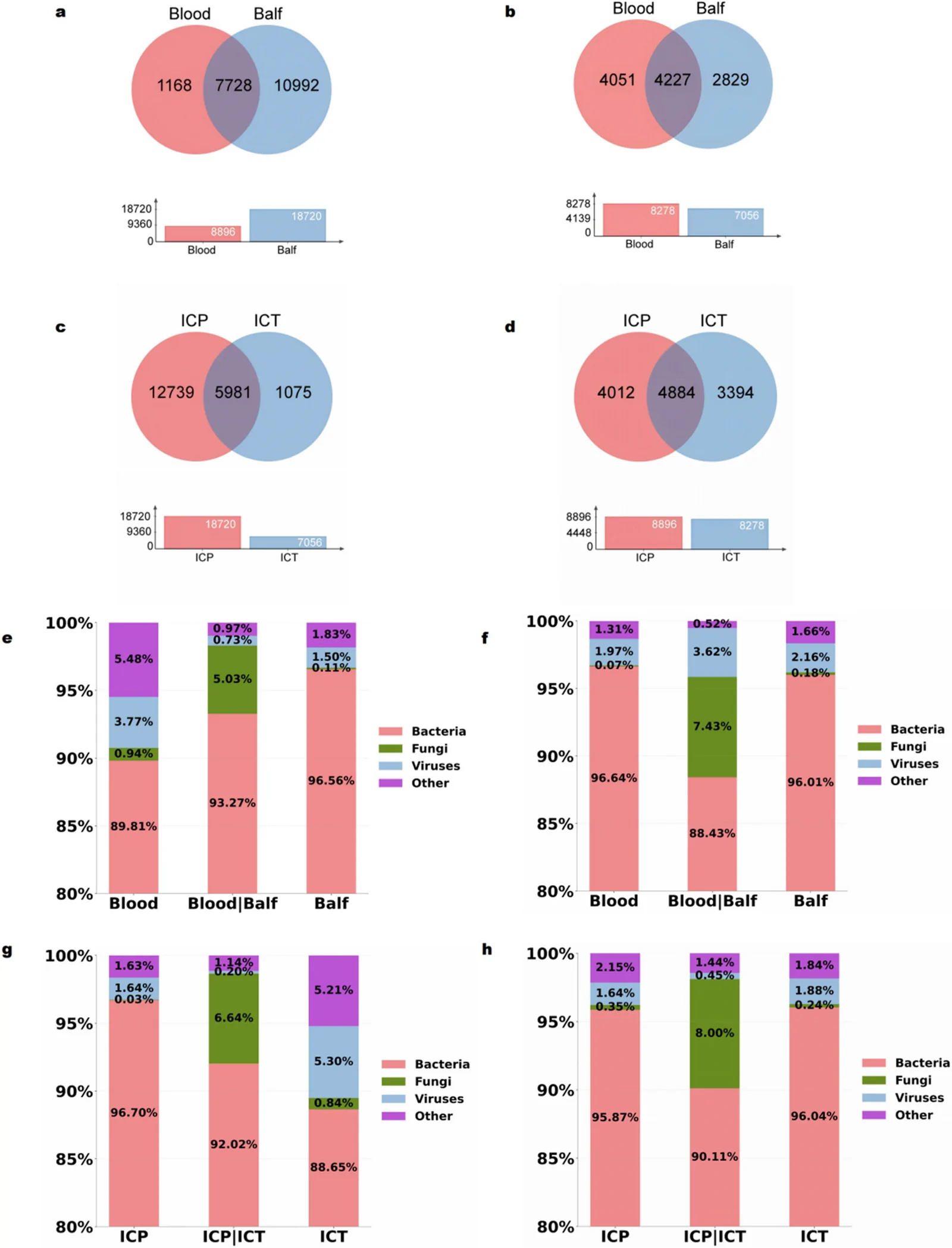
Comparison of Shared and Specific Microbial Composition in Blood and BALF. **a**-**b** The Venn diagrams display the number of specific and shared microbes at species level between blood and BALF: **a** from ICP patients; **b** from ICT patients. **c**-**d** The Venn diagrams display the number of specific and shared microbes at species level between patient groups: **c** BALF of ICP vs. ICT patients; **d** blood of ICP vs. ICT patients. **e-f** Stacked bar plots showing kingdom-level distribution of: **e** Blood-BALF specific and shared taxa in ICP cohort; **f** Blood-BALF specific and shared taxa in ICT cohort; **g** ICP-ICT differential and co-existed taxa in BALF; **h** ICP-ICT differential and coexisted taxa in blood

The number of specific and shared microbes in BALF and blood was further analyzed and illustrated using Venn diagrams (Fig. 4a-d). In total, 18,720 microbes were detected in the BALF and 8,896 microbes in the blood of ICP patients. Among these, 7,728 microbes were found in both BALF and blood, indicating that 86.87% of microbes identified in blood were shared with BALF. In contrast, only 7,056 microbes were detected in the BALF of ICT patients, which is less than half of the number found in the BALF of ICP patients, with 5,207 (73.80%) of those being consistent between the two groups (Fig. 4a and b). For blood samples, microbial counts were similar between groups (ICP: 8,896; ICT: 8,278), with 59.00% of blood microbes being shared. These results suggest that immune status may significantly impact the microbiome in the lower respiratory tract compared to blood. In addition, only 51.06% of blood microbes in ICT patients coexisted in BALF, significantly much lower than that (86.87%) overlap in ICP patients. This finding potentially reflects an increased permeability of the alveolar-capillary barrier in ICP patients, facilitating microbial DNA translocation from the lower respiratory tract into the bloodstream.

To investigate potential preferential release of specific microbial categories from lower respiratory tract into blood, we analyzed the composition of shared microbial taxa between BALF and blood at the kingdom level using bar charts (Fig. 4e-f). The results revealed that, compared to BALF-specific communities, the proportion of fungi increased substantially in the shared microbial communities, from 0.11% to 5.03% in ICP patients and from 0.07% to 7.43% in ICT patients, while the proportions of viruses and bacteria remained relatively stable or decreased. This disproportionate increase suggests that fungal DNA is more readily released from the lower respiratory tract into circulation than bacterial or viral DNA. This phenomenon may be explained by the larger genome size of fungi, which increases sequence concentration and enhances detection probability. This pattern was observed not only in ICP patients but also in ICT patients (Fig. 4e and f). Additionally, comparative analysis of microbial species showed that nearly all the fungi species detected in ICP patients were also present in ICT patients (Fig. 4g and h). It appears that the diversity of fungal species is more stable and less susceptible to the effects of immune status compared to bacteria and viruses.

### Screening blood microbes as indicators of their presence in BALF

Since more than half the microbes detected in the blood were also found in the BALF, this suggests that these microbes potentially largely originate from the lower respiratory tract. We sought to identify specific microbes whose presence in the blood strongly predicts their existence in BALF, which could facilitate pneumonia diagnosis when BALF sampling is impractical or high-risk. To generate a highly precise list of microbes, we established the following criteria: (1) the microbe must be detected in the blood in at least one sample; (2) at least 70% of the patients who had this microbe in their blood also showed its presence in their BALF. According to this standard, 2,060 microbes were screened in ICP patients, compared to only 501 in ICT patients (Supplementary Tables 9, 10). Among these, 210 microbes were shared between both groups. These results suggest that blood tests may be more suitable for pneumonia pathogen detection in ICP patients compared to ICT patients, consistent with the observed higher proportion of blood microbes coexisting in the lower respiratory tracts of this population.

### The differential gene expression in BALF between ICP and ICT patients

**Fig. 5:**
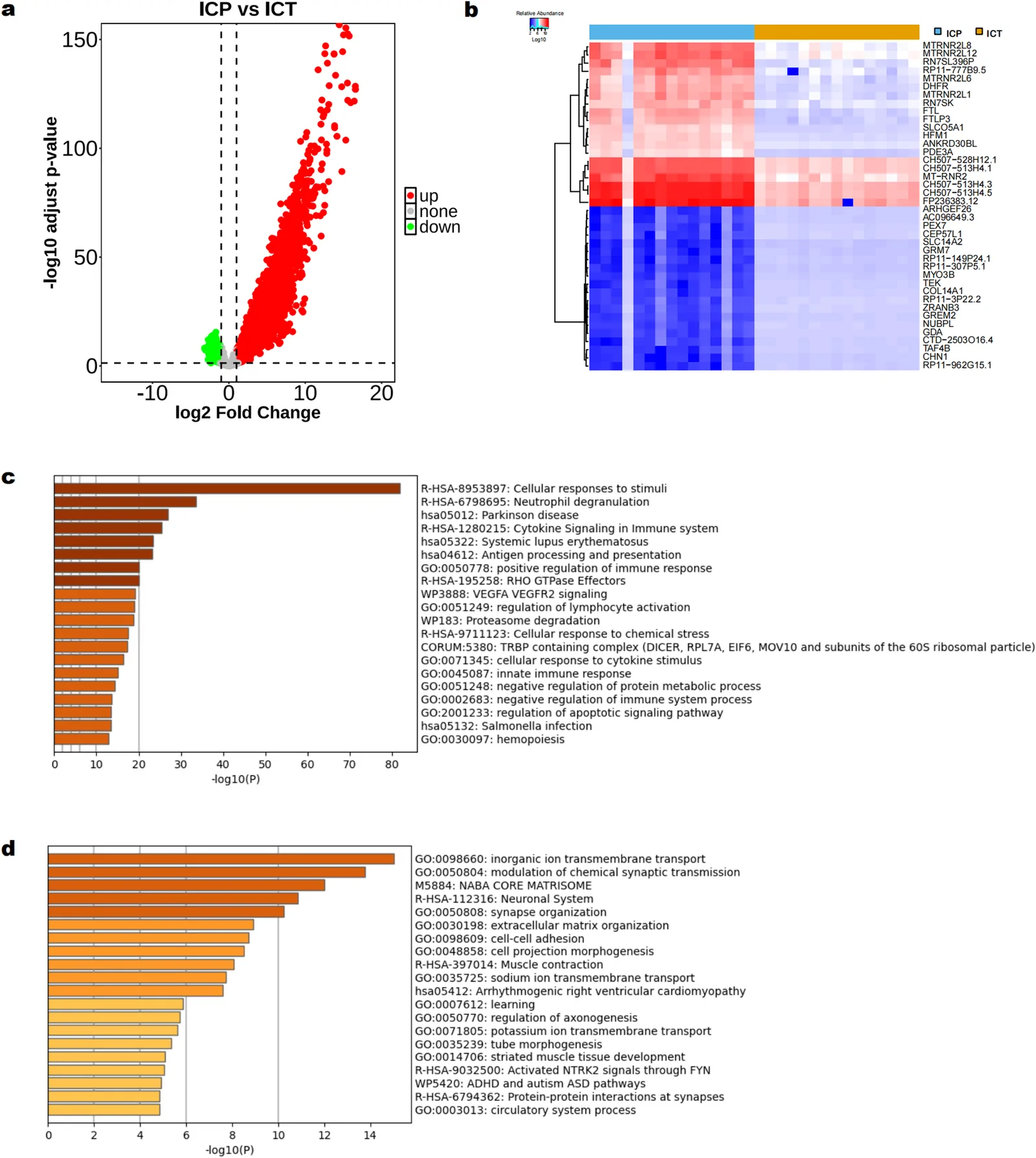
The Differential Gene Expression in BALF Between ICP and ICT Patients. **a** The volcano plot shows the differential gene expression in BALF between fifteen ICP and fifteen ICT patients. **b** The heatmap of the top 15 significantly upregulated and downregulated genes across individual patients, with hierarchical clustering based on expression z-scores. **c**-**d** The histographs display the enriched pathway and function of the top 1000 upregulated (**c**) and downregulated **d** genes

To explore whether the increased percentage of shared microbial sequences between blood and BALF in ICP patients versus ICT patients resulted from increased permeability of alveolar-capillary barrier, we analyzed the difference in BALF gene expression between 15 ICP and 15 ICT patients through DEseq2 analysis. The analysis revealed 6525 upregulated and 4883 downregulated genes in ICP compared to ICT patients (Fig. 5a, Supplementary Table 11). Heatmap visualization displayed expression level of the top 20 most significantly up- and down-regulated genes (ranked by *P* value, Fig. 5b). Functional enrichment analysis of the top 1000 upregulated genes identified pathways including cellular responses to stimuli, immune response, regulation of immune response, antigen processing and presentation (Fig. 5c, Supplementary Table 12), suggesting heightened immune activation against infection, inflammation, or immune disorders or tissue repair like angiogenesis in ICP patients. Additionally, the activity of these pathways may be associated with various pathological conditions such as autoimmune diseases, infections, or tumors. Conversely, the top 1000 downregulated genes were enriched in NABA CORE MATRISOME, extracellular matrix organization, and cell-cell adhesion (Fig. 5d, Supplementary Table 13), which were important for integrity of the alveolar-capillary barrier.

### Associations between microbial features and host gene expression

To determine potential links between alveolar-capillary barrier impairment and BALF microbial composition in ICP patients, we conducted Spearman correlation analysis (|ρ| ≥ 0.8, indicating strong relationships) between microbial abundance and host gene expression in four key pathways: NABA CORE MATRISOME, extracellular matrix organization, extracellular matrix degradation, and cell-cell adhesion. Results showed significant negative correlations between pathway genes and 27 microbial species, including *Rhizobiales bacterium*, *Acidovorax delafieldii*, *Xanthomonas arboricola*, and *Alcaligenes faecalis* (Supplementary Table 14). All these microbes were significantly enriched in ICP patients, suggesting a potential role in increasing permeability of respiratory barrier integrity. Notably, *Xanthomonas campestris* exhibited the most extensive gene correlations, suggesting a potential central role in ICP-associated barrier dysfunction.

### Validation of findings with an enlarged sample size and deeper sequencing

**Fig. 6:**
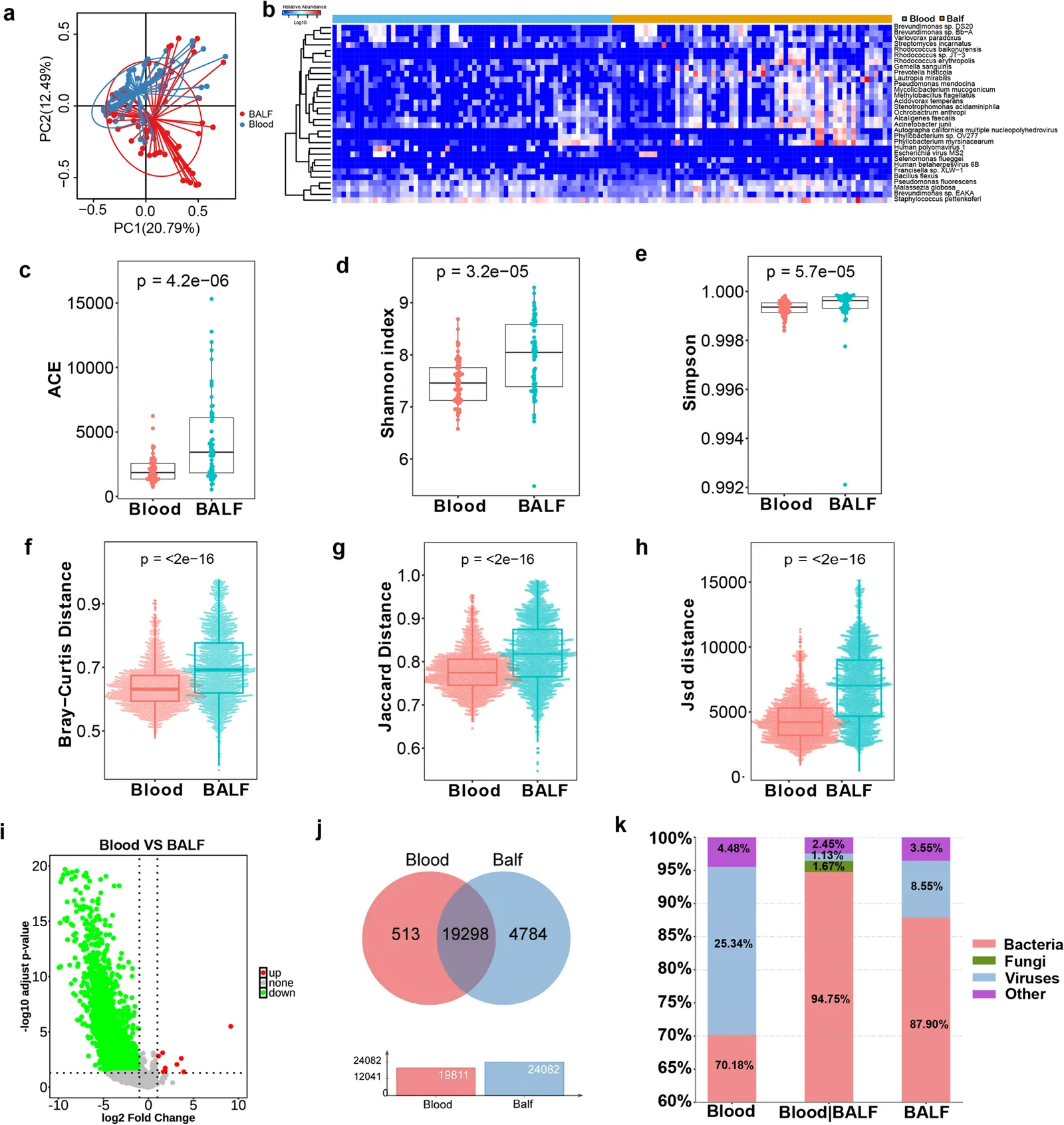
Validation of Findings with an Enlarged Sample Size (in 62 ICP patients) and Deeper Sequencing. **a** The PCA plot shows the overall differences in the microbial DNA sequences from blood and BALF samples of 62 ICP patients. The statistical significance in difference was examined by PERMANOVA. **b-d** The box plots display microbial alpha-diversity in BALF and blood in ICP patients, including ACE index (**b**), Shannon index (**c**) and Simpson index (**d**). **e**-**g** The box plots display microbial beta-diversity including Bray-Curtis distance (**e**), Jaccard distance (**f**) and Jensen-Shannon Distance (**g**). The statistical significance was analyzed by Wilcoxon signed-rank test. **h** The volcano plot indicate the differential abundance of specific microbial DNA sequences between blood and BALF samples from 62 ICP patients. **i** The heatmap show the abundance of the top 15 high and least microbes in blood compared to BALF in each ICP. **j** The Venn diagrams display the number of specific and shared microbes between BALF and blood in 62 ICP patients. **k** The bar charts illustrate the composition of coexisting and specific microbial taxa between BALF and blood at the kingdom level in 62 ICP patients

Validation in an expanded cohort of 62 immunodeficient pneumonia patients (with 10-fold increased sequencing depth and 21 patients from the initial cohort) confirmed our initial findings. Among the 62 patients, 36 (58.06%) were male and 26 (41.94%) were female, with a mean age of 38.98 years (± 14.08). Detailed baseline clinical characteristics are provided in Supplementary Table 1. Of the 62 total samples, 24 pathogens were definitively diagnosed in 53 samples (Supplementary Table 15). PCA showed distinct blood-BALF microbial diversity separation (Fig. 6a), with significantly higher alpha and beta diversity in BALF (Fig. 6b-g). A total of 4,593 differentially abundant microbes were identified in BALF compared to blood (Fig. 6h), exceeding those found at lower sequencing depths. Heatmap visualization showed relative abundance of the top 20 most and least abundant microbial taxa in BALF compared to blood (ranked by *P* value, Fig. 6i). These differentially abundant microbes showed strong association with autoimmune diseases, such as rheumatoid arthritis (Supplementary Fig. 3), corroborating earlier findings. Besides, total microbial counts increased to 24,082 (BALF) and 19,811 (blood), with 97.4% blood microbes shared with BALF (Fig. 6j). Fungal DNA demonstrated greater blood detectability than viral DNA, with all fungal species co-occurring in both compartments (Fig. 6k). In addition, based on the established criteria for screening microbes present in BALF, a total of 1,321 microbes were identified (Supplementary Table 16). This indicates that at least 70% of patients with a particular microbe in their blood also exhibited its presence in BALF. Nine final pathogens diagnosed in 20 samples appeared in the screened list. Four hundred and nineteen microbes were found to be consistent in the samples from the 21 ICP patients (Supplementary Table 17). The list contains at least 25 common respiratory pathogens (Supplementary Table 17 marked in yellow). Six final pathogens identified from the 62 ICP patients were present in this shared microbial list. These results confirmed the findings from the cohort of the 21 ICP patients. The proportion of fungi in the shared microbial list was 7.15%, which was much higher than that (0.11%, Fig. 4e) in the BALF specific microbial sequences, and the proportion of viruses in the shared microbial list was only 0.95%, supporting the preference in fungal markers predicting fungal infections.

## Discussion

Our paired analysis of BALF-blood microbial sequences in ICP and ICT patients revealed significant inter-compartmental diversity differences, particularly pronounced in ICP patients. The BALF of ICP individuals showed notably greater microbial diversity, including species potentially linked to autoimmune conditions like rheumatoid arthritis. Remarkably, we identified 20 differentially abundant microbial markers capable of distinguishing immune status with high accuracy (AUC = 0.91). The substantially greater sharing of microbial DNA sequences between blood and BALF in ICP patients (versus ICT) suggests potentially increased permeability of the alveolar-capillary barrier, which may facilitate microbial DNA translocation from respiratory tract to bloodstream. Importantly, we identified 419 blood-derived microbial signatures that reliably indicated lower respiratory tract presence, offering valuable diagnostic potential when BALF collection is impractical or risky.

As described above, ICP patients showed significantly higher percentages of non-human reads in BALF compared to blood, whereas the percentages remained similar in ICT patients. Moreover, the non-human reads in the BALF of ICP patients were four times higher than those in ICT patients, suggesting greater microbial burden in the lower respiratory tract. This elevated microbial load likely results from impaired immune defenses facilitating colonization by diverse microorganisms, particularly opportunistic pathogens - a finding consistent with existing literature [27]. Many enriched species in ICP patients represent known opportunistic pathogens, suggesting their potential utility as immune status biomarkers. However, the significant increase in alpha diversity observed in ICP patients compared to ICT patients differs from other studies. For instance, untreated HIV patients exhibit low alpha diversity and high beta diversity compared to normal controls, with specific species such as *Prevotella* and *Veillonella* being enriched [28, 29]. This discrepancy likely reflects fundamental differences in study design: our control group consisted of ICT pneumonia patients whose microbial profiles were already altered by infection, whereas HIV studies typically compare against healthy controls. Thus, our findings represent the combined impact of both immune status and active pulmonary infection on microbiome composition.

Besides, a significantly higher proportion of microbial DNA sequences in the blood was found to be shared with BALF in ICP patients compared to ICT patients. The human bloodstream does not harbor a stable microbiome [1], as microbial DNA in the blood is believed to originate from various body sites such as the gut, oral cavity, skin, and respiratory tract. To minimize potential confounding from gut-derived contamination, we removed gut-associated microbial reads from blood samples. Nevertheless, other sources (e.g., oral, skin, genitourinary) could still contribute. However, given that the concordance between blood and BALF microbes was substantially higher in ICP patients than in ICT patients (86.87% vs. 51.06%), it is plausible that a substantial portion of blood microbial DNA in ICP patients might originate from the lower respiratory tract. Consistent with this interpretation, we observed downregulation of genes involved in ECM organization and cell-cell adhesion, suggesting increased alveolar‑capillary permeability that could facilitate translocation of microbial DNA from the lung into the blood. The alveolar-capillary barrier consists of epithelial and endothelial cells that interact with each other across a fibrous extracellular matrix (ECM) [30], and hence the downregulation of cell-cell adhesion and ECM interaction can increase the barrier permeability. In addition, the upregulated genes in ICP patients were significantly enriched in pathways such as cellular responses to stimuli, neutrophil degranulation, cytokine signaling in immune system, antigen processing and presentation, immune response, regulation of immune response, and intracellular pathogen defense (e.g., salmonella infection pathway (hsa05132)). These findings reflect a more activated immune response and heightened inflammation within the alveolar space, indicative of the immune system’s effort to combat infection. Of note, BALF transcriptomics captures the local immune environment rather than systemic immune status. Consequently, persistent antigenic stimulation from lower respiratory tract infection—due to impaired host defense—may drive the increased recruitment and hyperactivation of immune cells at the local site, despite low systemic immune cell counts. As a result, this sustained cellular influx and hyperactivation can lead to excessive inflammation, which may further damage respiratory tissues and increase vascular permeability [31, 32]. Persistent immune activation and inflammation was commonly observed in immunodeficiency patients with HIV infection [33]. Upregulation of pathways like VEGFA VEGFR2 signaling and Rho GTPases has also been linked to the vascular permeability and leakage [31]. Together, these findings underscore the increased permeability of the alveolar-capillary barrier in ICP patients, explaining the higher proportion of shared microbial DNA sequences between blood and BALF in this group. Additionally, this observation aligns with the microbial profiles observed, indicating that a greater variety of microbes in ICP patients could potentially serve as indicators of their presence in BALF. It also suggests that using blood tests as a surrogate for BALF analysis is more feasible in ICP patients than in ICT patients. This is particularly significant, given that ICP patients often present in poorer health and may be unable to undergo invasive procedures.

Additionally, we observed that fungal DNA appeared more readily detectable in blood compared to viral sequences—a finding discordant with clinical observations where certain pathogens (e.g., cytomegalovirus [CMV]) were frequently identified in blood. This discrepancy may arise from two key factors: (1) Our analysis focuses on both pathogenic and non-pathogenic microbial DNA, whereas clinical diagnostics typically focus on targeted detection of specific pathogens; (2) Certain viruses (e.g., CMV) may evade host immunity and undergo active replication in blood, generating sufficient nucleic acid concentrations for detection.

Furthermore, this study generated two microbial lists with potential clinical applications of blood testing as a substitute for BALF testing in diagnosing pneumonia. If a microbe identified in blood samples through culture-based or molecular diagnostic methods appeared in these lists, this strongly suggests pulmonary presence of the pathogen, thereby supporting a possible diagnosis of pneumonia. To date, numerous studies [3–7] have compared the diagnostic performance of blood testing and BALF testing for identifying pathogens in pneumonia, with findings indicating that BALF generally performs better, and blood testing may have advantages in identifying viral infections. In cases of sepsis, high consistency has been observed, and clinical features have been investigated as markers to determine when blood can effectively serve as a substitute for BALF [8]. In contrast to previous studies, our research seeks to identify specific microbial indicators that can guide the use of blood testing as a surrogate for BALF analysis. This study presents the first comprehensive comparison of microbial communities (including both pathogenic and non-pathogenic species) between paired BALF and blood samples. Our findings reveal significant microbial overlaps between these two compartments, with taxonomic preferences observed among the shared species. Notably, these shared microbial signatures demonstrate potential diagnostic utility for pneumonia detection via blood-based assays. Furthermore, our data suggest that host immune status may influence these microbial distribution patterns. Due to the small sample size in this study, the lists have not been fully verified. However, the study provides a method to explore the conditions under which blood tests can be effectively used to diagnose lower respiratory tract infections. The significant differences observed between BALF and blood in ICP patients, but not in non-ICP patients, suggest that certain species may serve as biomarkers for evaluating immune status.

This study has several limitations. First, the modest cohort size (*n* = 21 per group) may underpower detection of rare pathogen, necessitating validation in larger multicenter trials. Second, our findings are derived exclusively from patients with post‑HSCT pneumonia and may not be directly generalizable to other immunocompromised populations, such as solid organ transplant recipients or patients receiving chemotherapy or biologics without a history of HSCT. Third, the lack of pathogen-specific sensitivity/specificity analysis (due to low sample numbers per species) precludes direct clinical application. Fourth, while we propose blood-BALF microbial sharing as a proxy for barrier integrity, this hypothesis requires direct histological or imaging confirmation in future studies. Fifth, our study lacked a control group of asymptomatic HSCT patients. Without such a comparator, we cannot determine whether the increased number of microbial signatures in blood compared to ICT patients is specifically driven by active pneumonia or merely reflects the baseline immunocompromised state. Future studies incorporating asymptomatic HSCT patients are needed to clarify this mechanistic question. Sixth, despite rigorous aseptic collection, we cannot fully exclude potential contamination from skin, oral, genitourinary or urinary sources - a known limitation of metagenomic studies. However, our requirement that blood-detected microbes also be present in BALF ensures that our predictive signatures are derived from concordant signals between the two compartments, supporting the potential utility of blood-based detection as a surrogate for BALF in identifying lower respiratory tract-associated microorganisms. Seventh, the strength of our blood‑BALF shared list lies in its ability to predict lower respiratory tract involvement when a marker is detected in blood. Nevertheless, the list should be used as a supportive tool alongside clinical evaluation, as it may not cover all respiratory pathogens.

## Supplementary Information


Supplementary Material 1.
Supplementary Material 2.


## Data Availability

The datasets used and/or analysed during the current study are available from the corresponding author on reasonable request.
