# Supplementary material for: Microbial DNA analysis of paired blood-bronchoalveolar lavage fluid in post-HSCT patients with pneumonia implying application conditions of blood as a surrogate in pathogen detection
Source: Respir Res. 2026 Jun 18;27:338. doi: 10.1186/s12931-026-03779-z (PMC13531842; doi:10.1186/s12931-026-03779-z)
Supplement: Supplementary file 1 [file 12931_2026_3779_MOESM2_ESM.docx]

**Supplementary figures**

**Fig. S1. Comparative analysis of microbial DNA composition and diversity in blood and BALF between ICP and ICT patients.**

(a-b) Principal component analysis (PCA) of microbial communities based on Bray-Curtis distances showing significant separation between 21 ICP and 21 ICT patients in (a) BALF and (b) blood samples (PERMANOVA).

(c-h) Box plots comparing alpha diversity between ICP and ICT patients: (c-e) ACE, Shannon, and Simpson indices in BALF samples; (f-h) Corresponding indices in blood samples. Significance determined by paired Wilcoxon signed-rank test.

(i-l) Beta diversity dissimilarity metrics: (i-j) Bray-Curtis and Jaccard distances for BALF samples; (k-l) Corresponding distances for blood samples. Center lines represent medians, boxes show IQR (paired Wilcoxon test).

**Fig. S2. Performance of the immune status prediction model.**

**Fig. S3. The association of upregulated microbes in BALF (ICP vs. ICT patients) with diseases according to the online tool MicrobiomeAnalyst.**
